# Neutralization of Microbiota-Derived Corisin Shows Early Amelioration of Advanced Pulmonary Fibrosis

**DOI:** 10.3390/arm94010009

**Published:** 2026-02-06

**Authors:** Kazuki Furuhashi, Hajime Fujimoto, Masaaki Toda, Corina N. D’Alessandro-Gabazza, Atsuro Takeshita, Kota Nishihama, Tomohito Okano, Haruko Saiki, Atsushi Tomaru, Valeria Fridman D’Alessandro, Isaac Cann, Esteban C. Gabazza, Taro Yasuma, Osamu Hataji, Tetsu Kobayashi

**Affiliations:** 1 Department of Pulmonary and Critical Care Medicine, Diabetes, Metabolism and Endocrinology, Faculty and Graduate School of Medicine, Mie University, Edobashi 2-174, Tsu 514-8507, Mie, Japan; k-furuhashi@med.mie-u.ac.jp (K.F.); genfujimoto1974@yahoo.co.jp (H.F.); atsurolennon@clin.medic.mie-u.ac.jp (A.T.); k-nishihama@clin.medic.mie-u.ac.jp (K.N.); okatomojin525@clin.medic.mie-u.ac.jp (T.O.); harusakusa@yahoo.co.jp (H.S.); a-tomaru@clin.medic.mie-u.ac.jp (A.T.); 2 Microbiome Research Center, Mie University, Edobashi 2-174, Tsu 514-8507, Mie, Japan; gabazza@med.mie-u.ac.jp (E.C.G.); t-yasuma0630@clin.medic.mie-u.ac.jp (T.Y.); 3 Department of Immunology, Faculty and Graduate School of Medicine, Mie University, Edobashi 2-174, Tsu 514-8507, Mie, Japan; t-masa@doc.medic.mie-u.ac.jp (M.T.); immunol@doc.medic.mie-u.ac.jp (V.F.D.); 4 Department of Animal Science, University of Illinois Urbana-Champaign, Urbana, IL 61801, USA; icann@illinois.edu; 5 Respiratory Center, Matsusaka Municipal Hospital, Tonomachi 1550, Matsusaka 515-8544, Mie, Japan; mch1031@city-hosp.matsusaka.mie.jp

**Keywords:** advanced lung fibrosis, microbiota, corisin, transforming growth factor-β1, apoptosis

## Abstract

**Highlights:**

**What are the main findings?**

- Anti-corisin monoclonal antibody therapy rapidly improves inflammatory and fibrotic endpoints in established pulmonary fibrosis.
- Corisin neutralization reduces epithelial cell injury and extracellular matrix accumulation in fibrotic lungs.

**What are the implications of the main findings?**

- Corisin represents a druggable microbiota-derived mediator sustaining fibrosis beyond disease initiation.
- Therapeutic targeting of corisin may complement or extend current treatments for progressive fibrotic lung disease.

**Abstract:**

Background: Corisin, a microbiota-derived proapoptotic peptide, has emerged as a key mediator of epithelial injury, inflammation, and acute exacerbation in fibrotic lung disease. Although acute corisin inhibition prevents exacerbations in experimental models, its therapeutic impact on established pulmonary fibrosis remains unclear. This study evaluated the short-term efficacy of corisin neutralization in advanced transforming growth factor-β1 (TGF-β1)-driven lung fibrosis. Methods: Male TGF-β1 transgenic mice with established fibrosis were allocated to computed tomography-matched groups and treated intraperitoneally with an anti-corisin monoclonal antibody (clone 21A) or control IgG every two days for one week. Bronchoalveolar lavage fluid (BALF) analysis, histopathology, assessment of apoptosis, Ashcroft scoring, and lung hydroxyproline quantification were performed on day 8. Results: Anti-corisin treatment significantly reduced BALF inflammatory cell counts, including macrophages and lymphocytes. Histological analyses demonstrated decreased alveolar epithelial apoptosis, reduced collagen deposition, and significantly lower Ashcroft fibrosis scores. Lung hydroxyproline content was also markedly decreased, indicating attenuation of extracellular matrix accumulation. Conclusions: Short-term neutralization of microbiota-derived corisin rapidly alleviates inflammation, epithelial injury, and fibrotic remodeling in advanced TGF-β1-induced pulmonary fibrosis. These findings identify corisin as an upstream driver of ongoing fibrogenesis and support its potential as a therapeutic target in progressive fibrotic lung disease.

## 1. Introduction

Idiopathic pulmonary fibrosis (IPF) is a devastating and progressive interstitial lung disease characterized by irreversible scarring of the lung parenchyma and gradual loss of pulmonary function [1]. The disease arises from an aberrant wound-healing response to repetitive alveolar epithelial injury, leading to fibroblast activation, excessive extracellular matrix deposition, and architectural distortion of the lung [1]. Despite the availability of antifibrotic agents such as nintedanib and pirfenidone, the overall prognosis remains poor: the median survival is only two to three years after diagnosis and approximately three to four months following acute exacerbation [2]. In recent years, the lung microbiome has emerged as a potential modulator of IPF pathogenesis. Several studies employing 16S rRNA gene sequencing have shown that patients with IPF harbor an increased bacterial burden and reduced microbial diversity in bronchoalveolar lavage fluid (BALF) compared with healthy controls [3]. Enrichment of genera such as *Streptococcus*, *Haemophilus*, *Veillonella*, and *Neisseria* has been correlated with accelerated disease progression and increased mortality [3]. Longitudinal analyses further demonstrate that higher bacterial load predicts acute exacerbations and poorer outcomes, suggesting that dysbiosis may actively contribute to the inflammatory and fibrotic microenvironment in the lung [4].

Recent studies have identified corisin, a microbiota-derived proapoptotic peptide, as a critical mediator of epithelial injury and acute exacerbation in fibrotic lung disease [5]. Corisin was initially characterized as a transglycosylase-derived fragment from *Staphylococcus nepalensis*, and both corisin-containing bacterial culture supernatants and synthetic corisin peptides have been shown to induce apoptosis in alveolar epithelial cells and exacerbate experimental pulmonary fibrosis [5]. Subsequent work expanded on these observations by isolating *Staphylococcus haemolyticus* strains from fibrotic lung tissue cultures that expressed IsaA-family transglycosylases containing corisin-like sequences. The peptides derived from these enzymes reproduced potent apoptotic activity not only in pulmonary epithelial cells but also in parenchymal cells of the skin, kidney, retina, and intestine [6]. Consistent with a broader pathogenic role, corisin-like motifs are evolutionarily conserved within transglycosylases from other clinically relevant bacteria, including *Listeria monocytogenes* and *Mycobacteroides abscessus* [6]. Mechanistically, these peptides are generated through cleavage by a secreted bacterial serine protease, yielding active fragments that trigger mitochondrial-dependent apoptotic signaling in host cells [6]. In vivo, intratracheal administration of corisin induces acute exacerbation of pulmonary fibrosis in both TGF-β1-overexpressing mice and bleomycin-induced fibrosis models [6]. Importantly, prior in vivo studies have focused primarily on the role of corisin in disease initiation or acute exacerbation settings, including preventive or exacerbation-triggering models. However, while corisin has been implicated in acute exacerbation in TGF-β1 transgenic mice that spontaneously develop lung fibrosis, whether corisin continues to actively drive fibrotic progression once fibrosis is established and whether it can be therapeutically targeted at this stage remain unknown.

Corisin acts rapidly on alveolar epithelial cells, initiating mitochondrial dysfunction, inflammatory activation, and early extracellular matrix deposition, pathogenic events that unfold over short time intervals and critically shape the trajectory of fibrogenesis [6,7]. These observations raise the possibility that corisin may function not only as a trigger of acute injury but also as a sustained upstream mediator of ongoing fibrotic remodeling. Given the highly dynamic nature of epithelial injury and fibroproliferative responses, therapeutic intervention after fibrosis establishment represents a stringent and clinically relevant test of disease modifiability [8]. Based on this rationale, we hypothesized that neutralization of microbiota-derived corisin using a monoclonal antibody, even over a brief treatment window, would ameliorate key pathological features of TGF-β1-driven pulmonary fibrosis. Accordingly, the present study was designed to evaluate whether short-term therapeutic administration of a neutralizing anti-corisin monoclonal antibody improves pathological hallmarks of advanced TGF-β1-induced lung fibrosis, including inflammatory cell infiltration, epithelial apoptosis, and extracellular matrix accumulation.

## 2. Materials and Methods

### 2.1. Animals

TGF-β1 transgenic (TG) mice on a C57BL/6J background, which spontaneously develop progressive and ultimately fatal pulmonary fibrosis, have been previously described [5]. The male TG mice used in this study weighed 23–26 g and were 8–10 weeks of age. Animals were bred and housed under specific pathogen-free conditions at 21 °C, with a 12-h light/dark cycle, in the Experimental Animal Facility at Mie University. Each cage was provided with wood-wool nesting material, and the mice had free access to standard chow and water. Genotyping of TG mice was performed by standard PCR analysis of genomic DNA extracted from tail biopsies using transgene-specific primer pairs [5].

### 2.2. CT Examination

Computed tomography (CT) of the lungs was performed using a micro-CT scanner (Latheta LCT-200; Hitachi Aloka Medical, Tokyo, Japan). Following induction of anesthesia with inhaled isoflurane, mice were positioned prone for image acquisition as previously described [5]. To enable standardized assessment of disease severity, the severity of pulmonary fibrosis in the TGF-β1-induced lung fibrosis model was assessed using a semiquantitative CT fibrosis scoring system, whereby increasing scores reflect progressive fibrotic remodeling of the lung parenchyma, with even-numbered scores representing intermediate stages between defined fibrotic grades, allowing finer discrimination of fibrosis severity, as follows: score 1, normal lung architecture; score 2, intermediate findings; score 3, mild fibrosis; score 4, intermediate findings; score 5, moderate fibrosis; score 6, intermediate findings; and score 7, advanced fibrosis [6].

### 2.3. Treatment of TGF-β1 Transgenic Mice with Anti-Corisin Monoclonal Antibody

**Figure 1:**
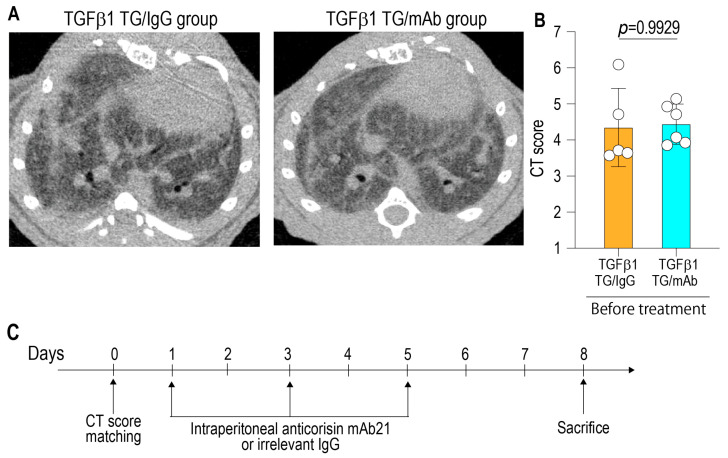
Experimental design and baseline chest CT score matching. (**A**) Representative axial chest computed tomography (CT) images of TGF-β1 transgenic (TG) mice assigned to the control IgG group (left) or the anti-corisin monoclonal antibody (mAb) group (right), demonstrating comparable baseline fibrotic changes. (**B**) Quantitative CT fibrosis scores before treatment, confirming no significant difference between groups (4.34 ± 1.08 vs. 4.44 ± 0.55; *p* = 0.9929). (**C**) Experimental timeline illustrating CT score matching at baseline (day 0), intraperitoneal administration of anti-corisin mAb (clone 21A) or irrelevant IgG every two days, and sacrifice on day 8. Data are presented as mean ± SD. Statistical comparisons were performed using a two-tailed unpaired Student’s *t*-test. TGFβ1 TG/IgG: Transforming growth factor-β1 transgenic mice treated with irrelevant IgG. TGFβ1 TG/mAb: Transforming growth factor-β1 transgenic mice treated with anticorisin monoclonal antibody.

A chest CT study was conducted in male TGF-β1 transgenic mice with established lung fibrosis, and the animals were allocated into two CT score-matched groups (Figure 1A,B). One group received intraperitoneal administration of the anti-corisin monoclonal antibody (clone 21A) (20 mg/kg of mouse weight), whereas the control group received an irrelevant IgG at the same dose. Antibody injections were administered every 2 days for a total of 3 doses over 1 week. All animals were euthanized on day 8 following the initial antibody administration (Figure 1C).

### 2.4. Biochemical Analysis

Collagen type I levels were quantified by enzyme immunoassay using an anti-collagen type I antibody and a biotin-conjugated anti-collagen type I antibody (Rockland Immunochemicals, Limerick, PA, USA). The hydroxyproline content of lung tissue was determined by a colorimetric assay (Hydroxyproline Colorimetric Assay Kit; BioVision, San Francisco, CA, USA) according to the manufacturer’s instructions. Corisin concentrations were measured by enzyme-linked immunosorbent assay (ELISA) as previously described, employing a polyclonal anti-transglycosylase 351 antibody as the capture antibody and a biotinylated anti-corisin 9A monoclonal antibody as the detection antibody [6]. Briefly, the capture antibody was coated onto 96-well plates at a final concentration of 2 μg/mL in phosphate-buffered saline (PBS) and incubated overnight at 4 °C. After blocking with 1% bovine serum albumin (BSA) in PBS and washing with PBS containing Tween-20, standard corisin solutions and plasma samples were added to the wells and incubated overnight at 4 °C. The plates were then washed and incubated with horseradish peroxidase-conjugated streptavidin (R&D Systems, Minneapolis, MN, USA). Following additional washing steps, color development was achieved by adding the substrate solution, and absorbance was measured at 450 nm. Corisin concentrations were calculated from a standard curve generated using known concentrations of recombinant corisin.

### 2.5. Bronchoalveolar Lavage Fluid

Mice were anesthetized for the collection of bronchoalveolar lavage fluid (BALF) used for biochemical and cytological analyses. Briefly, the trachea was cannulated with a 20-gauge needle, and the lungs were lavaged with sterile saline. The recovered lavage fluid was centrifuged, and the supernatants were stored at −80 °C until further analysis. The resulting cell pellets were resuspended in physiological saline for total and differential cell counts. Total cell numbers were determined using a nuclei counter (ChemoMetec, Allerød, Denmark), and cytospin preparations were stained with May–Grünwald–Giemsa (Merck, Darmstadt, Germany) for differential cell enumeration.

### 2.6. Collection of Lung Samples and Evaluation of Pulmonary Fibrosis

Mice were euthanized by an overdose of anesthesia, and the lungs were excised, fixed in 10% neutral-buffered formalin, embedded in paraffin, and sectioned for hematoxylin–eosin (H&E) staining. Histopathological evaluation was performed using an Olympus BX50 microscope equipped with Plan objectives and an Olympus DP70 digital camera (Tokyo, Japan). The severity of pulmonary fibrosis was assessed using the Ashcroft scoring system applied to H&E-stained sections and by quantifying lung hydroxyproline content via a colorimetric assay. For the Ashcroft score, microphotographs of five randomly selected microscopic fields from each lung were obtained; eight blinded observers independently graded the degree of fibrosis, and the mean fibrosis score for each mouse was calculated.

### 2.7. Statistical Analysis

Data are presented as the mean ± standard deviation (SD). Statistical comparisons between two groups were performed using a two-tailed unpaired or paired Student’s *t*-test, as appropriate. A *p*-value < 0.05 was considered statistically significant. All statistical analyses were conducted using GraphPad Prism version 10 (GraphPad Software, San Diego, CA, USA).

## 3. Results

### 3.1. Attenuation of Inflammation Following Corisin Neutralization

**Figure 2:**
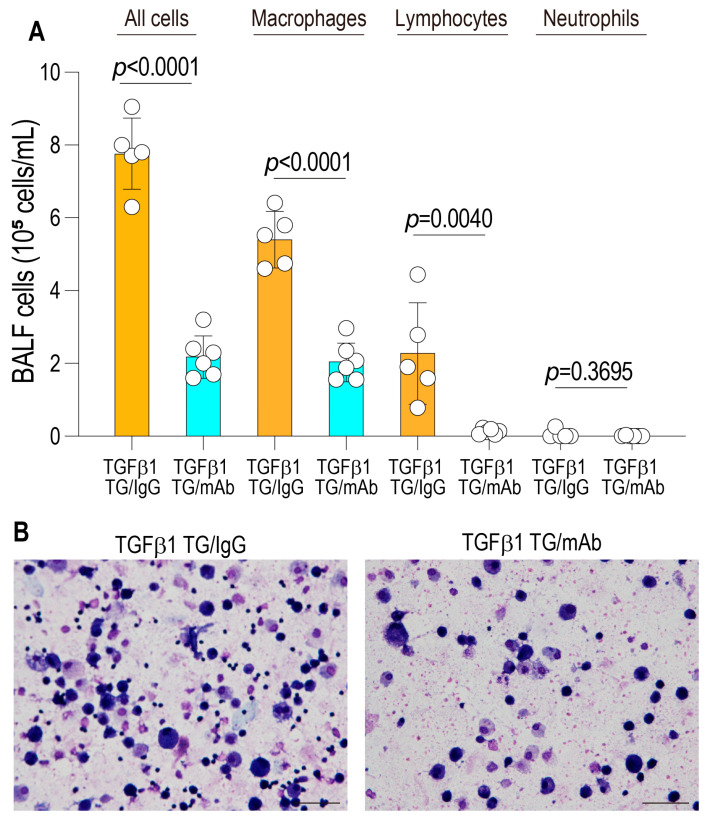
Amelioration of airway inflammation following corisin neutralization. (**A**) One group of TGF-β1 transgenic (TG) mice received intraperitoneal anti-corisin monoclonal antibody (mAb21), and the control group received irrelevant IgG. Bronchoalveolar lavage fluid (BALF) was collected on day 8, and inflammatory cell counts were quantified in TGF-β1 TG mice treated with anti-corisin mAb21 or irrelevant IgG. *n* = 5 for TGF-β1 TG/IgG and *n* = 6 for TGF-β1 TG/mAb. (**B**) Representative photomicrographs are shown. Scale bars = 50 µm. Data are expressed as the mean ± SD. Statistical analysis was performed using a two-tailed unpaired Student’s *t*-test. TGFβ1 TG/IgG: Transforming growth factor-β1 transgenic mice treated with irrelevant IgG. TGFβ1 TG/mAb: Transforming growth factor-β1 transgenic mice treated with anticorisin monoclonal antibody.

TGF-β1 transgenic mice with established lung fibrosis were treated with either an anti-corisin monoclonal antibody or an isotype-matched control IgG and euthanized on day 8 after the initial administration. BALF was collected, and inflammatory cell populations were quantitatively analyzed. Mice receiving anti-corisin treatment exhibited a significant reduction in total BALF inflammatory cell counts (7.77 ± 0.98 vs. 2.2 ± 0.58 [×10^5^ cells/mL]; *p* < 0.0001), with particularly pronounced decreases in macrophage (5.41 ± 0.74 vs. 2.06 ± 0.53 [×10^5^ cells/mL]; *p* < 0.0001) and lymphocyte (2.30 ± 1.39 vs. 0.12 ± 0.07 [×10^5^ cells/mL]; *p* = 0.0040) populations, compared with control IgG-treated animals (Figure 2A,B). These results indicate that short-term corisin neutralization effectively suppresses ongoing pulmonary inflammation in advanced TGF-β1-driven lung fibrosis.

### 3.2. Amelioration of Fibrotic Remodeling Following Corisin Neutralization

**Figure 3:**
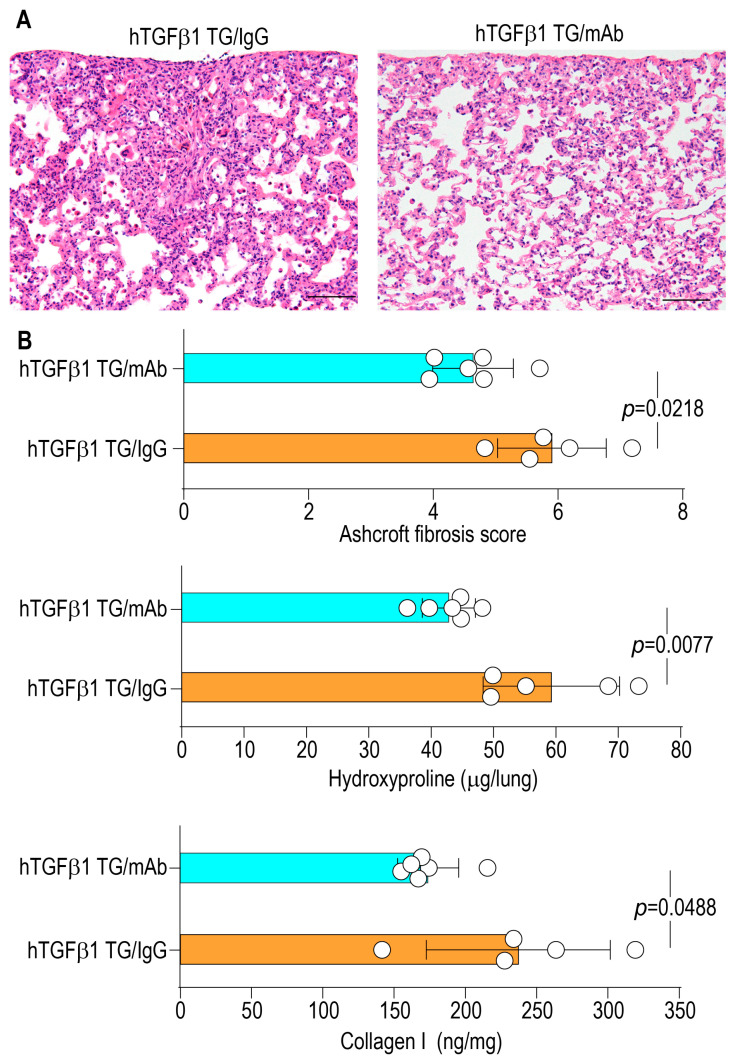
Attenuation of lung fibrosis after treatment with anti-corisin monoclonal antibody. (**A**,**B**) Lung tissues were stained with H&E, the severity of fibrosis was scored blindly using the Ashcroft scale, and lung hydroxyproline and collagen I levels were quantified. Representative photomicrographs are shown. Scale bars = 100 µm. *n* = 5 for TGF-β1 TG/IgG and *n* = 6 for TGF-β1 TG/mAb. Data are expressed as the mean ± SD. Statistical analysis was performed using two-tailed unpaired Student’s *t*-test. TGFβ1 TG/IgG: Transforming growth factor-β1 transgenic mice treated with irrelevant IgG. TGFβ1 TG/mAb: Transforming growth factor-β1 transgenic mice treated with anticorisin monoclonal antibody.

To assess the impact of corisin neutralization on fibrotic remodeling, lung tissues were subjected to hematoxylin and eosin (H&E) staining, and fibrosis severity was evaluated using the Ashcroft scoring system, in conjunction with quantitative measurements of lung hydroxyproline and collagen I content. Anti-corisin-treated mice demonstrated significantly lower Ashcroft scores (5.90 ± 0.87 vs. 4.63 ± 0.64; *p* = 0.0218) and markedly reduced levels of hydroxyproline (59.28 ± 10.93 vs. 42.83 ± 4.25 μg/lung) and collagen I (237.10 ± 64.53 vs. 173.90 ± 21.42 ng/mg; *p* = 0.0488) compared with mice treated with the irrelevant antibody, reflecting attenuated extracellular matrix accumulation (Figure 3A,B). Collectively, these findings demonstrate that short-term corisin neutralization not only mitigates inflammation but also attenuates fibrotic remodeling in advanced TGF-β1-induced pulmonary fibrosis.

### 3.3. Reduction in Epithelial Apoptosis and Corisin Burden Following Corisin Neutralization

**Figure 4:**
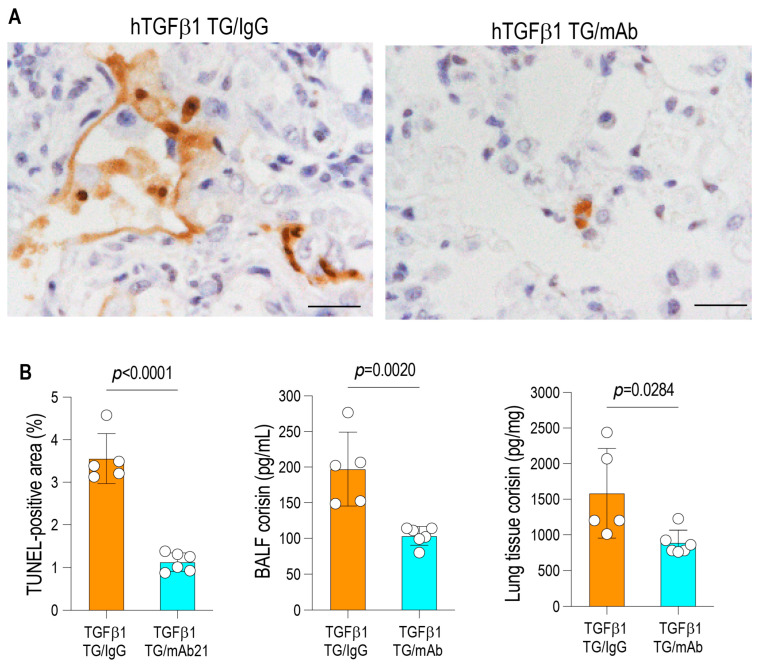
Reduction in apoptosis and corisin levels following corisin neutralization. (**A**,**B**) Apoptotic cells in lung tissue were identified by TUNEL staining, and the number of TUNEL-positive cells was quantified using WinRoof imaging software. Corisin concentrations in bronchoalveolar lavage fluid and lung tissue were measured by enzyme immunoassay. *n* = 5 for TGF-β1 TG/IgG and *n* = 6 for TGF-β1 TG/mAb. Representative photomicrographs are shown. Scale bars = 20 µm. Data are presented as the mean ± SD. Statistical analysis was performed using two-tailed unpaired Student’s *t*-test. TGFβ1 TG/IgG: Transforming growth factor-β1 transgenic mice treated with irrelevant IgG. TGFβ1 TG/mAb: Transforming growth factor-β1 transgenic mice treated with anticorisin monoclonal antibody.

Given the observed attenuation of inflammation and fibrotic remodeling, we next examined whether corisin neutralization also mitigated ongoing cellular injury within the fibrotic lung. Apoptosis was assessed by TUNEL staining of lung tissue on day 8 after treatment initiation. Mice treated with the anti-corisin monoclonal antibody exhibited a significant reduction in TUNEL-positive cells (3.55 ± 0.58 vs. 1.12 ± 0.21%; *p* < 0.0001) compared with control IgG-treated animals, indicating a marked suppression of alveolar epithelial apoptosis. In parallel, corisin concentrations were quantified in BALF and lung tissue homogenates. Anti-corisin therapy resulted in significantly decreased corisin levels in both BALF (197.20 ± 51.96 vs. 103.50 ± 13.08 pg/mL; *p* = 0.0020) and lung tissue (1584.0 ± 630.6 vs. 888.6 ± 175.9 pg/mL; *p* = 0.0284) compartments, demonstrating effective peptide neutralization and reduced corisin availability within the pulmonary microenvironment (Figure 4A,B).

Collectively, these findings indicate that short-term corisin neutralization suppresses epithelial apoptosis and reduces corisin burden in advanced TGF-β1-driven pulmonary fibrosis, thereby interrupting an upstream pathogenic axis linking epithelial injury to inflammation and fibrotic progression.

## 4. Discussion

This study demonstrates that short-term administration of a monoclonal antibody targeting the microbiota-derived peptide corisin induces rapid and multifaceted improvement in inflammatory, apoptotic, and fibrotic parameters in a TGF-β1 transgenic mouse model of advanced pulmonary fibrosis. Within a one-week therapeutic window, corisin neutralization markedly reduced bronchoalveolar inflammatory cell infiltration, alveolar epithelial apoptosis, lung hydroxyproline content, collagen I deposition, and Ashcroft fibrosis scores, indicating a potential therapeutic effect even in established disease.

Microbiome-derived factors are increasingly recognized as dynamic regulators of fibrotic lung disease, influencing immune activation, epithelial injury, and the amplification of fibrogenic pathways. Recent reviews demonstrate that both lung and gut microbial communities modulate disease severity and progression in ILD and pulmonary fibrosis [9,10,11]. Within this framework, the present results extend prior observations on corisin by demonstrating that its pathogenic influence is not limited to acute exacerbation but persists during the chronic phase of TGF-β1-driven fibrotic remodeling. The rapid therapeutic response observed here suggests that corisin functions as an ongoing epithelial stress signal that sustains inflammatory and fibrogenic signaling in established disease.

Alveolar epithelial cell injury and apoptosis are central pathogenic features of idiopathic pulmonary fibrosis and its acute exacerbations. Loss of epithelial integrity disrupts normal repair processes, promotes fibroblast activation, and accelerates extracellular matrix accumulation [8,12,13,14]. The marked reduction in epithelial apoptosis following corisin neutralization in this study supports the concept that continued epithelial injury remains a key driver of fibrotic progression even at advanced stages. Targeting an upstream, microbiota-derived mediator of epithelial apoptosis may therefore represent a complementary strategy to current antifibrotic approaches, which primarily act downstream on fibroblast activity.

While this study focused on histological and biochemical endpoints, these measures capture the core structural determinants of lung function. Reductions in collagen deposition and fibrotic severity scores are well-established surrogates of parenchymal remodeling and are consistently associated with improved lung mechanics in experimental models. The absence of direct physiological measurements represents a limitation; however, given the very short intervention window, functional recovery may reasonably lag behind the rapid resolution of epithelial injury and matrix remodeling. Longitudinal assessments incorporating lung function testing will be necessary to determine whether the early structural improvements observed here translate into sustained physiological benefit.

The durability of antifibrotic effects following treatment cessation is another important consideration. The intervention period was intentionally restricted to capture immediate biological responses in advanced fibrosis. Although post-treatment persistence was not assessed, the rapid attenuation of epithelial apoptosis, inflammation, and extracellular matrix accumulation is consistent with suppression of active fibrogenic signaling rather than delayed resolution of established scarring. Whether early corisin neutralization alone is sufficient to induce lasting remodeling, or whether sustained inhibition is required, remains to be determined and will require extended follow-up and alternative dosing strategies.

The specificity of corisin neutralization also merits consideration. The anti-corisin monoclonal antibody is designed to neutralize peptide activity and is not expected to exert direct antimicrobial effects or selectively alter microbial composition. Accordingly, the observed therapeutic effects are most parsimoniously attributed to functional inhibition of corisin. Nevertheless, indirect effects on host–microbiome interactions cannot be excluded, as attenuation of epithelial injury and inflammation may secondarily influence microbial ecology. Future studies incorporating microbiome profiling will be important to distinguish direct peptide-specific effects from host-mediated changes.

Several limitations of the present study should be acknowledged. First, all experiments were conducted exclusively in male mice, and potential sex-dependent differences in corisin biology or in the response to corisin neutralization were not evaluated. Given accumulating evidence that sex hormones and immune–fibrotic pathways can modulate pulmonary fibrosis progression and therapeutic responses, future studies incorporating both sexes will be necessary to fully define translational relevance. Second, therapeutic efficacy was demonstrated in a single TGF-β1 transgenic model, and the generalizability of these findings to other experimental fibrosis models remains uncertain. Finally, the absence of human validation data underscores that corisin neutralization should currently be regarded as a promising but still experimental therapeutic approach. Future studies incorporating additional disease models and validation in human samples, such as bronchoalveolar lavage fluid or lung tissue analyses, will be essential to establish the translational potential of targeting microbiota-derived corisin in pulmonary fibrosis.

## 5. Conclusions

In conclusion, this study provides proof-of-concept evidence that short-term neutralization of the microbiota-derived peptide corisin can rapidly attenuate key pathological features of advanced TGF-β1-driven pulmonary fibrosis, including inflammation, epithelial apoptosis, and extracellular matrix remodeling. These findings support a role for corisin as an upstream mechanistic contributor linking the lung microbiome to ongoing fibrogenesis and highlight its potential as an experimental therapeutic target in established fibrotic lung disease. Further studies incorporating additional disease models, extended follow-up, and validation in human samples will be required to determine the translational relevance and clinical feasibility of corisin-targeted interventions.

## 6. Patents

Esteban C. Gabazza and Tetsu Kobayashi are inventors on a patent covering the TGF-β1 transgenic mouse line used in this study. Corina N. D’Alessandro-Gabazza, Esteban C. Gabazza, and Isaac Cann have filed an invention disclosure related to the apoptotic peptides identified in this work and the anti-corisin monoclonal antibodies developed for the treatment of pulmonary fibrosis.

## Data Availability

All data supporting the findings of this study are available within the manuscript. Additional data generated and analyzed during the current study are available from the corresponding authors upon reasonable request.

## Abbreviations

The following abbreviations are used in this manuscript:

TGF-β1	Transforming growth factor-β1
TG	Transgenic
BALF	Bronchoalveolar lavage fluid
CT	Computed tomography
IPF	Idiopathic pulmonary fibrosis
BSA	Bovine serum albumin
PBS	Phosphate-buffered saline
ARRIVE	Animal Research: Reporting of In Vivo Experiments
